# The Determination of Melanoma Stage at Diagnosis

**DOI:** 10.1155/2010/839829

**Published:** 2010-06-28

**Authors:** John A. H. Lee

**Affiliations:** Fred Hutchinson Cancer Research Center, M4-B874, 1100 Fairview Avenue N, Seattle, WA 98109, USA

## Abstract

The rising proportion of melanomas diagnosed at an early pathologic stage is commonly ascribed to better public education. However in the US SEER program of cancer registration it has been found that the rates for *in situ* melanomas are closely related by a log linear relationship to the incidence of invasive melanomas and that this relationship is unrelated to calendar year or gender or patient age. This relationship is sufficiently strong to leave little room for other factors. The relationship may be different in populations with different melanoma rates and responses to them. It is suggested that the results are due to variations within populations of individual response to melanoma cell proliferation.

## 1. Introduction

Over recent decades in many prosperous white populations the incidence of malignant melanoma of the skin has been rising. Concomitant with this, there has been a shift towards earlier diagnosis, with a consequent improvement in prognosis. This has been ascribed by numerous authors to improved public and professional education [1–4]. 

In contrast, it has been shown, in the US population-based cancer registration system, that there is a systematic proportional relationship between incidence rates for *in situ* melanoma and invasive melanoma in males and females at all ages and over a long time period [5]. 

This paper explores the relationship between the incidence rate data for *in situ* and invasive melanoma further. It shows that the proportionate relationship between the *in situ *and invasive rates is very powerful and leaves room for only a small contribution by any diagnostic or other historical change. An explanation for the link could be found in variations between individuals in resistance to developing melanomas.

## 2. Data and Methods

The SEER (Statistics Epidemiology and End Results) Program of the U.S. National Cancer Institute (NCI) covers a group of nine geographic areas within the contiguous US that provide population-based cancer registration from 1975 to present. Data from the years 1975 to 2004 for malignant melanoma of the skin were downloaded from the SEER website [6]. 

This study was restricted to white people. The cases analyzed were restricted to first diagnoses and were all histologically confirmed. Cases were classified at the time of diagnosis as *in situ* (limited to the epidermis), localized invasive, regional spread, and distant spread. The localized, regional, and distant cases were combined as invasive. The cases unstaged were not analyzed further.

The age range for both males and females was 15–84 in 14 five-year- age groups. 

The association between incidence rate of in situ and of invasive melanoma is modeled by Poisson maximum-likelihood regression. Goodness-of-fit of each model was assessed by a pseudo-R-squared measure that compared a model with just the intercept to a model with intercept and coefficient [7].

The data were tabulated using SEER Stat software [8] and analyzed using Stata 9.0. [9].

## 3. Results

**Figure 1:**
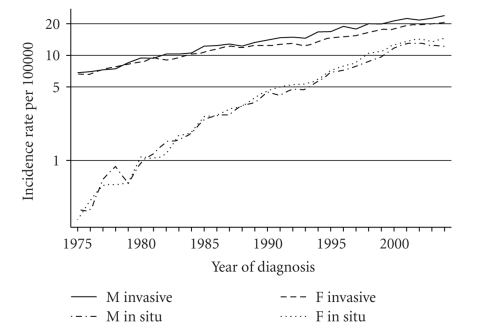
Rates at the time of diagnosis for invasive and *in situ* melanoma by single calendar years, US SEER program.

Rates for males and females for single years 1975–2004 and the age range 15–84 are shown for *in situ* and invasive melanoma for the nine geographic SEER areas combined in Figure 1. The rates are similar in males and females, while, as expected, over time the *in situ* rates rise much faster than the invasive. 

In Table 1 log linear models are shown for males and females relating the *in situ* and invasive rates for all discrete populations, defined by gender, age from 15 to 84 and period, from 1975–79 to 2000–2004. The confidence limits are very close and the coefficients of variation are close to one.

**Figure 2:**
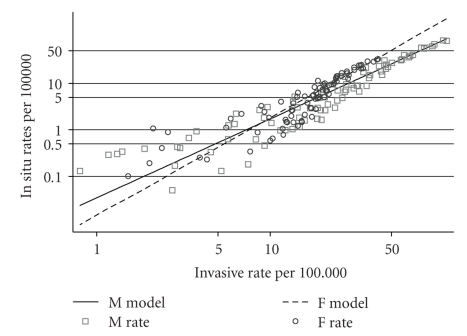
*In situ* rates at diagnosis plotted against invasive rates at diagnosis for six five-year-time periods (1975–2004) and fourteen five year age groups (15–190–80–84), and linear models (Table 1) (Males and Females, SEER [6]).

The models and rates are shown in Figure 2.

Similar models are also shown in the table for the separate 5-year time periods from 1975–1979 to 2000–2004. The coefficients decline slowly, apparently as the *in situ* rates approach a maximum (Figure 1). The coefficients of the period specific models for each sex are highly unlikely to be due to chance.

**Figure 3:**
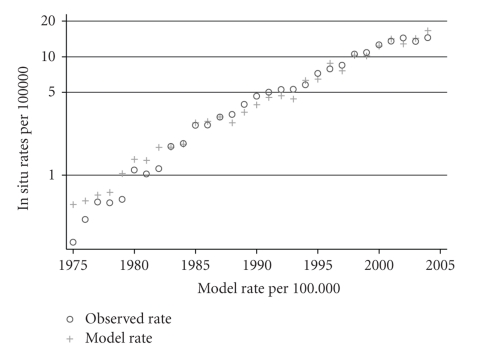
Comparison between observed rates in males for *in situ* melanoma and model rates derived from the single log linear equation for the whole period (Table 1) giving *in situ* rates derived from invasive rates for single years 1975–2004.

Figure 3 shows the set of 84 observed rates for *in situ* melanoma and estimated rates derived from the single two-parameter log linear equation and the set of observed invasive rates for males. The picture for females is similar. This is a demonstration (not a test, as there is only one data set) of the power of the relationship and its indifference to time or age.

## 4. Discussion

The connection between log incidence and log invasive rate for malignant melanoma is a substantial feature of the disease. The real distinction maybe between the tumors that have failed to penetrate into the dermis and those that have. But the precise value of the relationship may be an artifact produced by the original choice invasive rates as the independent variable and in situ rates as the dependent. For instance, a cutting point within the scale of Breslow thickness might be even better. 

 It is probable that resistance to the multiplication of malignant melanoma cells varies between people, and the systematic relationship between *in situ* and invasive rates reflects this. In a benign environment, those with the poorest resistance will be the only ones to get a melanoma and will present with the most advanced disease; in a severe environment, stronger resistors will get the disease, but be able to keep it *in situ*.

The data reported here are combined from nine SEER geographic populations scattered the United States from Detroit to Hawaii. Their separate examination could be helpful. It is possible that in other populations, such as those of Australia and New Zealand with different melanoma rates and different responses, other relationships will be found.

## Figures and Tables

**Table 1:** Parameters of linear models fitted to ln. rates for *in situ *and invasive melanomas. The parameters are shown for males and females for the entire period (84 data pairs for each gender) and for each period separately (14 data pairs).

Gender	Period	Coefficient	ucl	lcl	Constant	ucl	lcl	Pseudo *R* ^2^
Male	1975–2004	1.700	1.722	1.677	−14.891	−14.806	−14.977	0.940
Female	1975–2004	2.097	2.138	2.055	−15.760	−15.627	−15.893	0.867

Male	1975–79	2.275	2.607	1.944	−17.358	−16.415	−18.300	0.844
Male	1980–84	1.777	1.947	1.607	−15.708	−15.168	−16.248	0.868
Male	1985–89	1.702	1.796	1.609	−15.101	−14.775	−15.427	0.935
Male	1990–94	1.594	1.659	1.529	−14.537	−14.298	−14.777	0.966
Male	1995–99	1.472	1.516	1.427	−13.937	−13.762	−14.112	0.980
Male	2000–04	1.378	1.412	1.344	−13.419	−13.282	−13.557	0.986

Female	1975–79	1.766	2.186	1.347	−15.750	−15.627	−15.893	0.578
Female	1980–84	1.96	2.257	1.662	−15.944	−15.116	−16.772	0.703
Female	1985–89	1.733	1.914	1.552	−14.905	−14.375	−15.434	0.783
Female	1990–94	1.613	1.734	1.491	−14.247	−13.882	−14.613	0.873
Female	1995–99	1.542	1.631	1.453	−13.880	−13.592	−14.168	0.906
Female	2000–04	1.492	1.563	1.421	−13.571	−13.332	−13.809	0.951
